# Quality by Design-Based Scale-Up and Industrial Development of Turmeric Extract-Loaded Nanostructured Lipid Carriers

**DOI:** 10.3390/pharmaceutics18040492

**Published:** 2026-04-16

**Authors:** Wipanan Jandang, Phennapha Saokham, Chidchanok Prathumwon, Siriporn Okonogi, Chadarat Ampasavate

**Affiliations:** 1 Office of Research Administration, Chiang Mai University, Chiang Mai 50200, Thailand; wipanan.jandang@cmu.ac.th; 2 Department of Pharmaceutical Sciences, Faculty of Pharmacy, Chiang Mai University, Chiang Mai 50200, Thailand; phennapha.s@cmu.ac.th (P.S.); chidchanok.pra@dpu.ac.th (C.P.); siriporn.okonogi@cmu.ac.th (S.O.); 3 Center for Excellence in Pharmaceutical Nanotechnology, Faculty of Pharmacy, Chiang Mai University, Chiang Mai 50200, Thailand

**Keywords:** industrial production, scale-up, Quality by Design (QbD), high-shear homogenization, high-pressure homogenization, nanostructured lipid carriers (NLCs), turmeric

## Abstract

**Background/Objectives**: A robust and scalable manufacturing framework for lipid-based nanocarriers remains a critical challenge, particularly for labile phytochemicals such as curcuminoids in turmeric. This study presents an integrated Quality by Design (QbD)-driven and Outcome-Based Design (ObD) strategy to establish a scalable, resource-efficient manufacturing process for curcuminoids-loaded nanostructured lipid carriers (NLCs). **Methods**: To overcome the limitations of conventional multivariate design of experiments (DOE), which require extensive experimental runs, a risk-based, knowledge-driven single-factor screening approach was employed. Guided by risk assessment tools, including Ishikawa diagrams and failure mode considerations, 12 representative processing conditions were selected to define the design space. Critical quality attributes (CQAs), namely, particle size, polydispersity index (PDI), and zeta potential, were predefined to establish a robust control strategy. A two-step homogenization process—high-shear homogenization (HSH) for pre-emulsification followed by high-pressure homogenization (HPH) for nanoscale refinement—was systematically optimized. **Results**: Multivariate data analysis using principal component analysis (PCA) and hierarchical cluster analysis (HCA) identified key critical process parameters (CPPs), particularly HSH speed, processing time, and HPH cycles, as dominant factors influencing nanoparticle characteristics. The optimized 1-h process enabled successful scale-up of NLCs from 100 g to 5000 g, demonstrating the capability to generate nanosized particles within 100–500 nm. The combined HSH–HPH approach produced smaller, more uniform nanoparticles with high encapsulation efficiency and physical stability, outperforming HSH alone. **Conclusions**: Overall, this study establishes a practical and industrially viable framework that integrates QbD principles with data-driven optimization tools, for enabling reliable translation from laboratories to semi-industrial production.

## 1. Introduction

The development and scale-up of nanostructured lipid carriers (NLCs) from laboratory-scale to industrial-scale production remains a critical challenge in modern pharmaceutical manufacturing. Although NLC-based formulations have demonstrated promising physicochemical properties and biological performance at the laboratory level, their successful translation to large-scale production is often hindered by differences in processing conditions, equipment performance, and process reproducibility. Variations in shear force, mixing efficiency, heat transfer, and homogenization dynamics during scale-up can significantly alter critical quality attributes (CQAs), including particle size, polydispersity index (PDI), zeta potential, and encapsulation efficiency [1,2]. These inconsistencies may ultimately compromise product performance, stability, and manufacturability, thereby limiting successful commercialization.

Nanostructured lipid carriers (NLCs) have emerged as a promising lipid-based delivery platform due to their biocompatibility, ability to enhance drug solubility and stability, and suitability for controlled release [3,4,5]. In addition, NLCs are considered more favorable for industrial production compared with other nanocarrier systems, as they can be prepared using scalable techniques such as high-shear homogenization (HSH) and high-pressure homogenization (HPH) [3,6,7]. However, despite these advantages, the scale-up of NLC production still requires careful optimization to ensure that the physicochemical properties achieved at the laboratory scale can be consistently reproduced at larger production volumes [2,6,7].

A key limitation in industrial-scale development is the limited practicality of trial-and-error approaches or design of experiments (DOE), as these strategies often require a large number of production batches. Each large-scale production requires substantial quantities of active pharmaceutical ingredients and excipients, leading to high material costs, increased waste generation, and complex waste management. These constraints make extensive formulation screening and process optimization at the industrial level impractical. Consequently, laboratory-scale studies are essential for identifying optimal formulations and critical process parameters prior to scale-up. Nevertheless, even when similar processing techniques are employed, differences in equipment efficiency and process dynamics between laboratory and industrial systems can result in significant deviations in product quality [1,8].

Curcuminoids derived from turmeric (*Curcuma longa* L.) are well-established therapeutic agents and are widely utilized as model compounds to address key challenges in pharmaceutical development, including poor aqueous solubility, limited physicochemical stability, and low bioavailability [9,10,11,12]. Nano-delivery systems have demonstrated significant potential in overcoming these limitations, resulting in enhanced performance of bioactives at low concentrations while still achieving therapeutically relevant outcomes [2,3,12]. These considerations highlight the importance of rational formulation design at the laboratory scale, alongside the development of robust and scalable production strategies.

To address the challenges associated with scale-up, systematic approaches such as Quality by Design (QbD) have been increasingly adopted to facilitate process understanding and control. QbD emphasizes the identification of critical process parameters (CPPs) and their relationship with CQAs, enabling the development of robust and reproducible manufacturing processes [2,13,14,15]. This approach is particularly important for nanotechnology-based systems, where multiple interdependent variables can influence final product characteristics [16,17,18,19,20]. Therefore, this study focuses on the development and scale-up of NLC formulations from laboratory to industrial production. By applying QbD principles, a systematic evaluation of critical process parameters affecting the scale-up process was conducted. Curcumin was employed as a model compound representing high-value natural bioactives with limited availability. This study further examines whether the optimized laboratory-scale formulation can be successfully translated to larger production volumes while maintaining the desired critical quality attributes (CQAs). Ultimately, this work provides a practical framework for scalable NLC manufacturing and supports the industrial application of NLCs for drug delivery, minimizing variability during scale-up and ensuring consistent product quality across different production scales.

## 2. Materials and Methods

### 2.1. Materials

Polysorbate 80, sorbitan oleate, sweet almond oil, avocado oil, moringa oil, sunflower oil, olive oil, and turmeric extract (curcuminoids > 95%) were acquired from Welltech Biotechnology (Bangkok, Thailand). Curcumin standard (98%) was procured from MedChemExpress (Monmouth Junction, NJ, USA). Ethanol, methanol and acetonitrile were acquired from RCI Labscan (Bangkok, Thailand). Cetostearyl alcohol was acquired from Phitsanuchemicals (Phitsanulok, Thailand). Compritol 888 CG ATO (Glyceryl behenate) was obtained from Gattefossé (Paris, France). Ortho-phosphoric acid was acquired from Merck Millipore (Berlin, Germany).

### 2.2. Methods

#### 2.2.1. Analysis of Curcumin Using High-Performance Liquid Chromatography (HPLC)

Turmeric extract was analyzed to determine the amount of curcumin using the Agilent 1260 Infinity (Santa Clara, CA, USA). The turmeric extract was prepared using ethanol. The reverse-phase chromatography gradient elution was modified and simultaneously used. Curcumin as a marker compound was analyzed using the previous HPLC conditions with slight modifications [21]. Chromatographic separation was achieved in a Purospher^®^ STAR C-18 analytical column (RP-18 end-capped; 150 mm × 4.6 mm) (Merck KGaA, Darmstadt, Germany), with a gradient elution of 0.1% *v*/*v* phosphoric acid in water, acetonitrile and methanol. The flow rate was 1 mL/min. The samples were detected using a wavelength of 425 nm. This method was used for calculating the percentages of curcumin in the turmeric extract, photostability, percent entrapment efficiency (%EE), and percent loading capacity (%LC) of curcumin loaded in NLCs.

#### 2.2.2. NLC Formulations

Pre-liminary lab-scale formulations and production from our previous study were employed for the scale-up [3]. Oils with antioxidant and sun protection activity were evaluated for the protection of photo-induced degradation of curcumin following the experiment of Jandang et al. (2024) [12] according to the photostability testing of new active substances and medicinal products following the ICH Q1B guidelines [22].

Curcumin in various oils, namely, olive oil, sunflower oil, sweet almond oil, moringa oil and almond oil, was prepared in ethanol [3]. The sample mixtures in ethanol were exposed to UV and white light at 6000 to 7000 lux in the light chamber for 0, 4 and 8 h, with adjustments for the duration. At each time-point, the samples were collected and analyzed for the remaining amount of the marker compound using HPLC. The sample volume was adjusted to 2 mL before analyzing using HPLC. The log decrease in the extract concentration versus the time change was used to calculate the rate constant (K), as described by Jandang et al. (2024) [12].

(1) $$-\mathrm{Kt}=I\eta \;\frac{{\left[A\right]}_{t}}{{\left[A\right]}_{0}}$$

where [A]_t_ is the concentration at each time, [A]_0_ is the concentration at time 0 and K is the first-order rate constant.

Curcumin was degraded by first-order kinetics. Therefore, a rate constant was used to calculate the half-life (T_1/2_) of capsaicin and curcumin, as shown below:

(2) $$T_{1/2}\;=\;\frac{0.693}{K}$$

#### 2.2.3. Design Space and Control Strategy for Preparation of Curcuminoid-Loaded NLCs

The production of curcuminoid-loaded nanostructured lipid carriers (NLCs) was systematically scaled up from laboratory to medium-scale manufacturing through the application of a structured Quality by Design (QbD) framework. Critical process knowledge derived from our prior studies [3,23] was utilized to define the design space, within which key input variables were identified and controlled to ensure product quality in terms of predefined critical quality attributes (CQAs) [6,7,13].

To facilitate the transition to medium-scale production, design space development and control strategy were implemented through four sequential steps, as outlined below.

Step 1: Defining the quality target product profile (QTPP) and critical quality attributes (CQAs) of the curcuminoid-loaded NLCs.

The quality target product profile (QTPP) was defined based on the desired characteristics of a topical preparation containing curcumin-loaded nanostructured lipid carriers (NLCs). The target product was intended for dermal application with enhanced stability, controlled drug release, and improved therapeutic performance [24]. The key QTPP attributes of the NLCs included the following:

1. Physiochemical properties: Uniform nanoparticles with a particle size in the range of 100 nm to 500 nm, a polydispersity index (PDI) between 0.2 and 0.6, and a zeta potential ranging from −25 mV to −40 mV.
2. Entrapment efficiency: In the range of 75–100%.
3. Stability: Physically and chemically stable during storage, with no phase separation or significant degradation of curcumin.

Based on the defined QTPP, the critical quality attributes (CQAs) of the curcuminoid-loaded NLCs, such as particle size, polydispersity index, zeta potential, and entrapment efficiency, were subsequently identified to ensure that the final product met the intended quality, stability, and therapeutic efficacy.

Step 2: Identifying critical material attributes (CMAs).

Critical material attributes (CMAs) refer to the properties of raw materials that can significantly influence CQAs. In this study, the functional roles of each component and their impact on CQAs were considered as follows:

Solid lipids: Compritol 888 ATO (glyceryl behenate) and cetostearyl alcohol were chosen for their mixed internal crystal structure, promoting high drug loading. The differences in melting points, crystallinity, and hydrophobicity influenced the particle size, entrapment efficiency, and matrix stability of the nanoparticles.

Liquid lipid: Potential oils, i.e., olive oil, sunflower oil, sweet almond oil, moringa oil and avocado oil, were studied for improving the stability and solubility of curcuminoids, resulting in entrapment efficiency enhancement (EE%).

Surfactants: Polysorbate 80 and sorbitan oleate were selected for the emulsion formation. Particle size and nanoparticle aggregation prevention were monitored during the optimization. Their hydrophilic–lipophilic balance (HLB) was adjusted to ensure uniform nanosized particles.

By defining CMAs in relation to their functional role, a clear link between materials and CQAs was established.

Step 3: Identifying critical process parameters (CPPs).

Critical process parameters (CPPs), namely, shear speed, time and homogenization cycles, significantly impact product quality, safety, and performance. If these parameters change outside the controlled range, the product may not meet specifications. The process variables have a significant impact on CQAs. The NLCs were prepared via a hot homogenization technique, which included the following steps:

1. Pre-emulsion formation: The temperature of the lipid melt and aqueous phase, stirring speed, and mixing time were varied to identify factors affecting initial droplet size and homogeneity.
2. High-shear homogenization (HSH): Shear speed (rpm) and time were determined to create droplet size reduction before being subjected to high-pressure homogenization processing.
3. High-pressure homogenization (HPH): Homogenization cycles and applied pressure were included to target the nanosize and uniform nanoparticles.
4. Process limitations associated with the operational constraints of the equipment: The maximum achievable shear rate of the high-shear homogenizer was 20,000 rpm, and the upper operating pressure of the semi-industrial high-pressure homogenizer was 200 bar. Furthermore, the processing duration was meticulously regulated to mitigate heat accumulation, which could otherwise accelerate compound degradation.
5. Cooling/solidification: The cooling rate and final temperature influence lipid crystallinity and long-term stability. The curcuminoid-loaded NLCs were cooled to room temperature.

The CPPs were carefully monitored and optimized to ensure consistent CQAs across batches.

Step 4: Risk assessment and establishing design space and control strategy.

**Figure 1:**
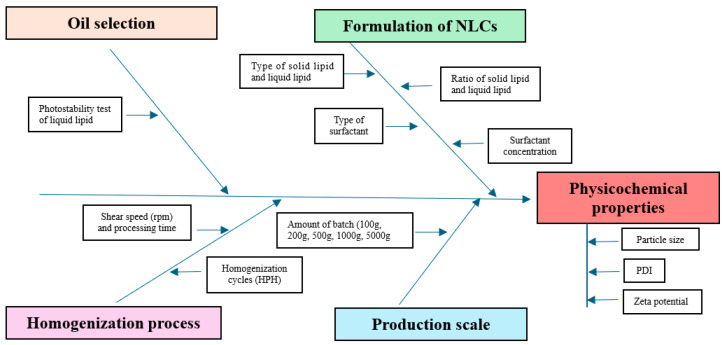
Ishikawa (Fishbone) diagram for quality by design in the modified scale-up of nanoparticle systems.

An Ishikawa (fishbone) diagram (Figure 1) was constructed to assess risks that could potentially impact the critical quality attributes (CQAs) of the curcuminoid-loaded nanostructured lipid carriers (NLCs), particularly during scale-up. Principal component analysis (PCA) and hierarchical cluster analysis (HCA) [13,25] were applied to the dataset from systematic single-factor screening, including the shear speed and processing time during HSH, as well as the pressure and cycles during HPH. This systematic approach was used to identify and categorize material attributes and process parameters that could influence product quality.

#### 2.2.4. Development of NLCs

From the design space, input variables that may influence critical quality attributes (CQAs), particularly particle size, PDI, and zeta potential, were adjusted from the initial lab scale (100 g), as shown in Table 1, Table 2 and Table 3. Stepwise increment scales (100, 200, 500, and 1000 g) of curcuminoid–NLC production were included, and the effects of each variable on CQAs were evaluated. A two-step homogenization strategy was employed, consisting of high-shear homogenization alone at speeds of 10,000 to 19,000 rpm for 10 to 20 min (HSH; T-25 Ultra-Turrax, Berlin, Germany) for laboratory-scale and high-shear homogenization, followed by high-pressure homogenization at 4 to 16 cycles (HPH; JG-1A, Shanghai, China, for 100–1000 g batch sizes and C and N Auto Machine, Samut Sakhon, Thailand, for a medium scale, 5000 g). The key process in preparing the pre-emulsions involved melting the fats at 75 °C and the water phase at 80 °C. Then the water phase was added to the oil phase, stirring by hand for 1 min before using HSH for mixing. Finally, after mixing was complete, the mixture was allowed to cool to room temperature. Next, high-pressure homogenization (HPH) was performed using 2, 3, 4, 8, or 16 cycles to achieve the target particle characteristics. The main components of the NLCs were solid lipids (glyceryl behenate and cetostearyl alcohol), liquid lipid (the selected oil), emulsifiers (polysorbate 80 and sorbitan oleate), and distilled water. The particle size, polydispersity index (PDI) and zeta potential closest to the predetermined (lab-scale) values were selected. The particle size, PDI, and zeta potential of the NLCs were measured using a zetasizer (Malvern, London, UK). The experiments were repeated in triplicate [26].

#### 2.2.5. Multivariate Analysis

A multivariate analysis was applied to recognize the influence on the physicochemical properties of the NLCs by the variation in the CPPs, namely, the process parameters of homogenization. Hierarchical cluster analysis (HCA) and principal component analysis (PCA) were performed using JMP Pro 18 (SAS Institute Inc., Cary, NC, USA) to explore patterns, similarities, and differences among the samples. HCA and PCA were applied after evaluating the particle size, polydispersity index and zeta potential of the NLCs. HCA and PCA were performed on the dataset comprising 12 different formulations. The lipid content and HPH condition (i.e., speed, time and cycle) during the NLCs’ preparation were defined as predictors. In the HCA, the distance between clusters was computed using the complete linkage method. PCA models were established through the row-wise estimation method and the correlation matrix. Components with eigenvalues greater than 1.0 were extracted as principal components (PCs) to explain the observed variations. Subsequently, the contributions of each independent and dependent variable were elucidated through the first two principal components (PC1 and PC2), highlighting the variables that influence the discrimination of formulations. The scores and loadings depicted in the biplot illustrated the coefficients of CPPs and CQAs across the two principal components. CPPs with an average factor loading score exceeding 0.3 were deemed critical for the variation observed in CQAs.

#### 2.2.6. Method Transfer for Medium-Scale NLC Production and Determination of %Entrapment Efficiency (EE) and %Loading Capacity (LC) of Curcuminoid-Loaded NLCs

Following the identification of critical process parameters (CPPs) at the 1000 g (pilot-scale) level, process transfer for scale-up to a 5000 g (medium-scale) batch was implemented using manufacturing-relevant homogenization equipment representative of industrial production conditions. The overall manufacturing procedure was maintained consistently with the pilot-scale processes, with the exception of adjustments to the homogenization cycle to accommodate the increased production volume.

The curcumin entrapment efficiency (%EE) and loading capacity (%LC) in the NLCs were determined using an indirect analytical approach. Briefly, 500 µL of the curcuminoid-loaded NLC dispersion was diluted with 4500 µL of ethanol in a centrifuge tube to obtain the total drug content, followed by centrifugation at 7000 rpm for 10 min at ambient temperature. In parallel, 500 µL of the NLC dispersion was transferred into an Amicon^®^ ultrafiltration tube (Merck KGaA, Darmstadt, Germany) and centrifuged at 3000 rpm for 60 min at room temperature to separate the unencapsulated fraction. The resulting filtrate, representing the free (non-entrapped) curcumin, was subsequently quantified using an HPLC-UV chromatograph.

The %EE and %LC were then calculated based on the difference between the total and free drug contents, and comparative analysis was performed between the 1000 g and 5000 g production batches using the equations provided below [12].

(3) $$\%\mathrm{EE}=\frac{Total\;compound\;added-Free\;(\text{non-entrapped})\;compound\times 100}{Total\;compound\;added}$$

(4) $$\%\mathrm{LC}=\frac{Weight\;of\;curcumin\;in\;lipid\;nanoparticles\times 100}{Weight\;of\;lipid\;nanoparticles}$$

#### 2.2.7. Statistical Analysis

Statistical analysis was calculated by a paired-sample *t*-test and one-way analysis of variance, with statistical significance at *p* < 0.01 and *p* < 0.05. All analyses were conducted using SPSS Program version 17.0.

## 3. Results and Discussion

### 3.1. Percentages of Curcumin in Turmeric Extract by HPLC-UV

The concentration of curcumin in the curcuminoid-rich turmeric extract and corresponding nanoformulations was quantified using a previously developed and validated HPLC-UV analytical method [21]. The turmeric extract contained 62.58% (*w*/*w*) curcumin. This quantified curcumin content was subsequently used as the basis for calculating the amount of active compounds incorporated into the lipid phase, nanoformulations, and production batches, as well as for determining entrapment efficiency (%EE), loading capacity (%LC), and stability profiles.

### 3.2. Photostability Test of Curcumin in Natural Oils

**Figure 2:**
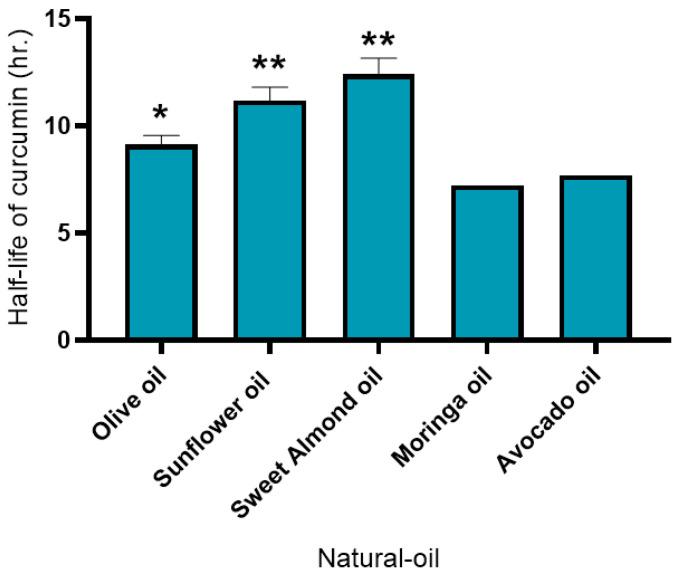
Half-lives of curcumin in 5 natural oils. The stability of curcumin in the selected oils was monitored in triplicate. Significant differences compared with data obtained with the treatment of all samples are identified with * *p* < 0.05 and ** *p* < 0.01, respectively, calculated using one-way ANOVA from the SPSS program.

Photostability evaluation was conducted using five natural oils: olive oil, sunflower oil, sweet almond oil, moringa oil, and avocado oil. Analysis of the curcumin content in the turmeric extracts incorporated into each oil showed significant differences when compared with the data obtained from the experiment with all samples. Moringa oil and avocado oil showed no significant difference, but moringa oil compared with olive oil showed a 95% (*p* < 0.05) significant difference, while sweet almond oil and sunflower oil showed a 99% (*p* < 0.01) significant difference, as shown in Figure 2. Notably, sweet almond oil has been previously reported to possess photoprotective properties, contributing to protection against UV-induced premature skin aging, which may partly explain its superior ability to stabilize curcumin under light exposure [27].

### 3.3. Effect of High-Shear and High-Pressure Homogenization on Curcuminoid-Loaded NLCs’ Physicochemical Properties

#### 3.3.1. Effect of Shear Speed

**Figure 3:**
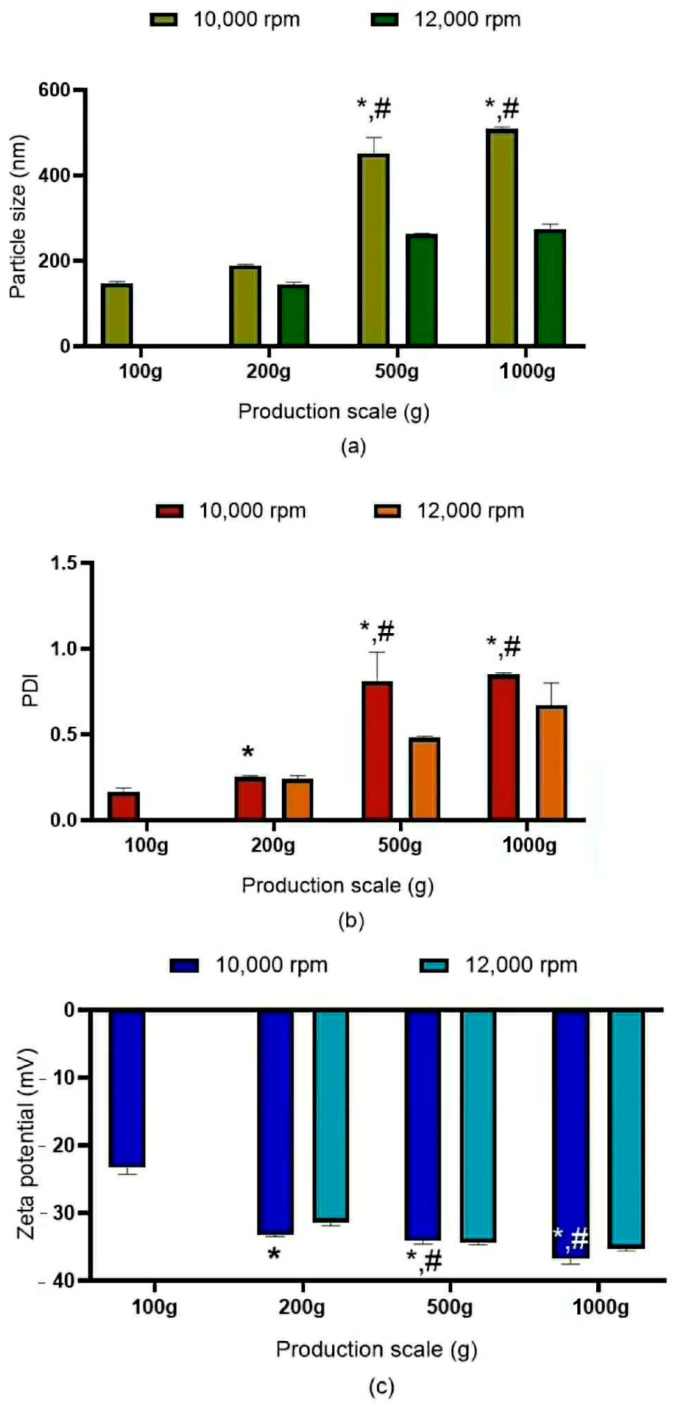
Influence of NLC preparation volume on high shear homogenization at 10,000 and 12,000 rpm for 10 min. The subpanels of the figure show (**a**) particle size, (**b**) particle size variety index (PDI), and (**c**) zeta potential. All experiments were performed with 3 replicates. * The value is significantly different when production scales are increased (*p* < 0.05). # The value is significantly different between shear speeds 10,000 rpm and 12,000 rpm at production scales of 500 g and 1000 g (*p* < 0.05). Significant differences were calculated using a paired *t*-test with the SPSS program.

The outcomes of developing NLCs from laboratory-scale to medium- and large-scale production rely on a Quality by Design (QbD) approach. To achieve these results, as illustrated in Figure 3, multivariate analysis was employed to identify the influence of critical process parameters (CPPs), particularly the high-pressure homogenization (HPH) process parameters, on the physicochemical properties of the NLCs. In the first multivariate factor, which involved increasing batch volumes, scale-up initiated from the use of high-shear homogenization (HSH) alone demonstrated volume-dependent effects. When two different HSH shear speeds were applied during scale-up, it was observed that at 10,000 rpm, increasing the batch volume from 100 g resulted in a corresponding increase in particle size. This shows that the same methods prepared in the laboratory cannot be applied to higher production scales. In contrast, at 12,000 rpm, the particle sizes obtained at 500 g and 1000 g did not differ significantly (*p* < 0.05). These findings indicate that increasing shear speed contributes to reduced particle size and leads to lower PDI values, resulting in more homogeneous formulations [28].

#### 3.3.2. Multifactor Analysis of Process Conditions

##### Effects of Shear Speed and Number of Homogenizing Cycles on Particle Size, PDI and Zeta Potential

**Figure 4:**
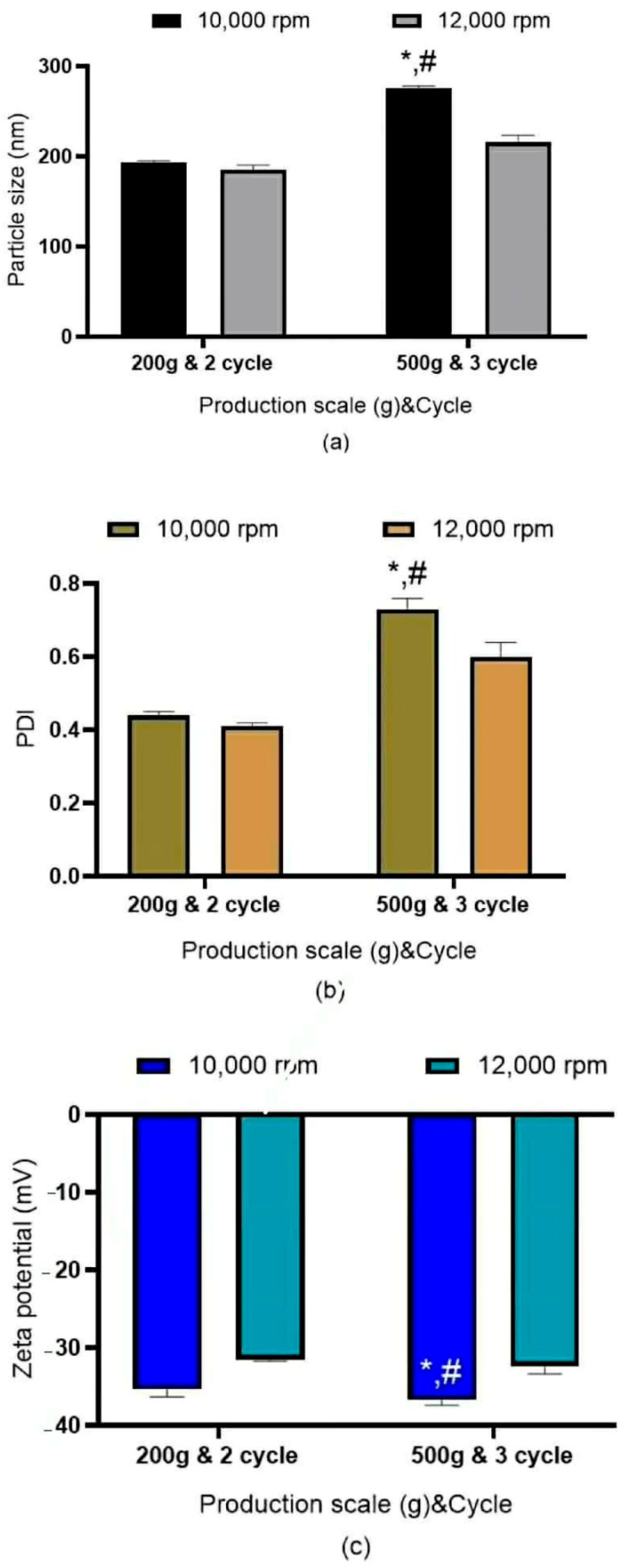
Effect of the NLCs’ preparation when both HSH and HPH were combined to produce 200 g and 500 g of NLC suspension. Sub-figure panels represent (**a**) particle size, (**b**) polydispersity index (PDI), and (**c**) zeta potential. The shear speed of HSH was between 10,000 and 12,000 rpm at a fixed processing time of 10 min. * The values are significantly different when production scales are increased from 200 g to 500 g (*p* < 0.05). # The value is significantly different between shear speeds of 10,000 rpm and 12,000 rpm (*p* < 0.05). Significant differences were calculated using a paired *t*-test with the SPSS program.

Figure 4 depicts the influence of HSH at 10 min and subsequent HPH cycles (two or three) on NLC production at scales of 200 g and 500 g. The findings indicate that conditions optimized for small-scale production markedly impacted nanoparticle characteristics when applied to larger batch sizes, particularly leading to increased particle size and broader size distribution. Notably, elevating the shear speed and the number of homogenization cycles resulted in a significant reduction in particle size (*p* < 0.05).

##### Effect of HSH Homogenization Time on the 500 g NLC Production Scale

**Figure 5:**
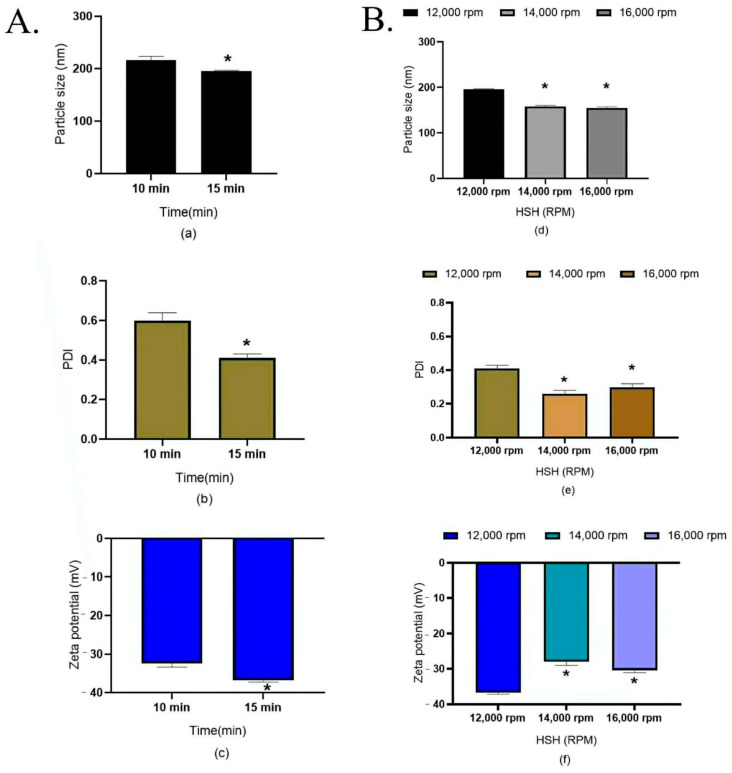
Effects of high-shear homogenization (HSH) conditions on NLC properties. (**A**) Effect of homogenization time at a fixed shear speed of 12,000 rpm. (**B**) Effect of shear speed (rpm) during preparation (500 g batch) using HSH for 15 min, followed by 3 cycles of high-pressure homogenization (HPH) at 250 bar. Sub-figure panels represent measured parameters: (**a**,**d**) particle size, (**b**,**e**) polydispersity index (PDI), and (**c**,**f**) zeta potential. All experiments were performed in triplicate. Asterisk (*) is significantly different at *p* < 0.05, calculated using a paired *t*-test with the SPSS program in (**A**). Asterisk (*) is significantly different at *p* < 0.05, calculated using one-way ANOVA from the SPSS program in (**B**).

As shown in Figure 5A, after defining the shear speed and high-pressure homogenization (HPH) conditions for the 500 g NLC production scale, the influence of the pre-emulsification time was investigated at a fixed shear rate of 12,000 rpm and three HPH cycles at 250 bar. As illustrated in Figure 5, increasing the homogenization time from 10 to 15 min led to a significant reduction in the particle size, polydispersity index (PDI), and zeta potential (*p* < 0.05). These results are consistent with previously reported trends, indicating improved droplet disruption and particle uniformity with prolonged mechanical input [29,30,31]. Nevertheless, extended processing may increase heat generation, which may promote degradation of thermosensitive active compounds. Therefore, the homogenization time represents a critical process parameter that must be balanced between product quality and thermal risk during scale-up.

At the 500 g NLC production scale, pre-emulsification at 12,000 rpm for 15 min produced nanoparticles with an average size of approximately 200 nm, which was larger than that obtained at the 100 g laboratory scale (~150 nm). This scale-dependent increase suggests that a higher shear intensity may be required for large-scale production. Accordingly, the effect of shear speed was evaluated (Figure 5B). Increasing the homogenization speed to 14,000 and 16,000 rpm for 15 min, followed by three HPH cycles, did not result in a statistically significant difference in particle size (*p* < 0.05). Although the smallest particle size was observed at 16,000 rpm, the marginal improvement indicates that further increases in shear speed do not necessarily provide proportional benefits for NLC production.

##### Effects of HSH Time (min) and Shear Speed (rpm) and HPH Cycles on the 1000 g NLC Production Scale

**Figure 6:**
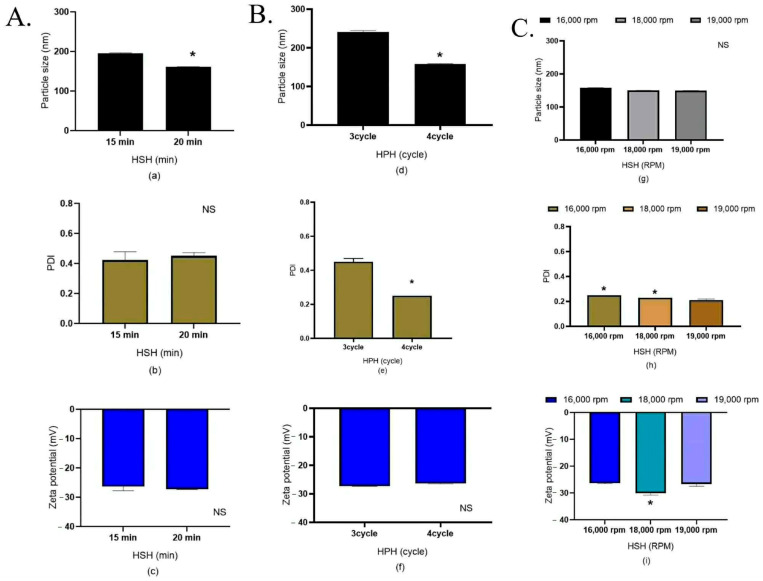
Effects of processing parameters on NLC properties (1000 g production scale). (**A**) Effect of HSH processing time. (**B**) Effect of number of HPH cycles. (**C**) Effect of shear speed (rpm) during HSH at a fixed speed of 16,000 rpm. Sub-figure panels represent measured parameters: (**a**,**d**,**g**) particle size, (**b**,**e**,**h**) polydispersity index (PDI), and (**c**,**f**,**i**) zeta potential. All experiments were performed in triplicate. Asterisk (*) is significantly different at *p* < 0.05, calculated using a paired *t*-test with the SPSS program in (**A**,**B**). Asterisk (*) is significantly different at *p* < 0.05, calculated using one-way ANOVA from the SPSS program in (**C**). NS indicates non-significant differences (paired *t*-test, SPSS).

In the subsequent pilot- or medium-scale production at 1000 g, to gain the NLC sizes of ~150 nm, the HSH time and HPH cycles were varied to determine whether the time and cycle of homogenization had a measurable impact on particle size reduction. The results (Figure 6A) showed that extending the processing time led to a significantly smaller particle size (*p* < 0.05). Similarly, the particle size reduction was effectively enhanced by increasing the HPH cycles (Figure 6B), confirming the influence of processing homogenizing time as a critical process parameter during scale-up. During scale-up from 200 g to 500 g and 1000 g, the high-pressure homogenization (HPH) pressure was constrained to 250 bar to align with the operational limitations of the semi-industrial HPH system (approximately 3000 psi or ~206.84 bar), thereby necessitating optimization of alternative process parameters when pressure cannot be further increased. Adjustment of the shear speed to 19,000 rpm in combination with four HPH cycles successfully produced NLCs that met the predefined quality attributes (Figure 6C). These findings demonstrate that preservation of laboratory-scale NLC characteristics during industrial-scale production requires the coordinated optimization of shear speed, processing time, and homogenization cycles rather than reliance on pressure intensification alone.

**Figure 7:**
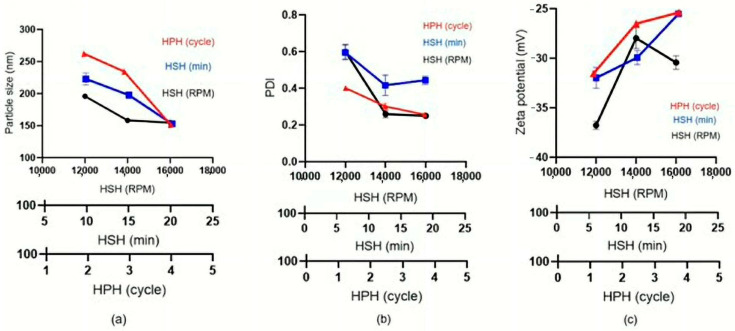
Effects of combined HSH and HPH conditions on the properties of curcuminoid-loaded NLCs at a production scale of 1000 g. Sub-figure panels represent (**a**) particle size, (**b**) polydispersity index (PDI), and (**c**) zeta potential.

As illustrated in Figure 7a, the number of homogenization cycles and the HSH processing time exerted significant influence on particle size, while the shear speed also contributed measurably. This trend is consistent with the zeta potential analysis in Figure 7c, in which all investigated parameters exhibited significant effects. The zeta potentials were observed in the range of (−25)–(−35) mV. In contrast, the particle size distribution (PDI) was predominantly governed by shear speed, with a comparatively lower contribution from homogenization cycles and HSH processing time (Figure 7b). This highlights the importance of strategically tuning the selected multiple CPPs during scale-up to maintain product quality and ensure consistent NLC characteristics across production scales. The pressure was maintained at 250 bar to closely approximate the pressure of high-pressure industrial homogenizers in the manufacturing plants.

High-shear and high-pressure homogenization are the kinds of high-energy approaches used for reducing the inner phase of emulsions since the 1950s to produce emulsions. The first publications about the use of HPH for large-scale production of lipid nanoparticles date back to around 2000 [2]. Medium-scale SLN production using HPH was developed at a throughput of 60 kg/h in a continuous homogenization mode [28,31]. Stepwise increases in processing capacities, ranging from laboratory-scale (~40 g) to medium-scale (~10 kg) and, further, to large-scale production (20–60 kg), were reported for the successful scale-up. The authors emphasized that maintaining similar homogenization principles and adequately controlling key process parameters during scale-up are essential to preserve particle characteristics, thereby supporting the feasibility of homogenization-based processes for reproducible large-scale production of lipid nanoparticles [32]. The mechanism of high-pressure homogenizers (HPH) or mixers applies high pressure to a liquid to force it through a membrane or valve that has narrow slits to effectively reduce the particle or droplet size. The process causes high shear, large pressure drops, and cavitation that homogenize the sample. In contrast, a high-shear homogenizer (or high-shear mixer) primarily operates based on rapid rotor–stator mixing to promote polydispersity and emulsification. As described in the literature, high-shear mixing mainly serves to achieve macroscopic homogeneity or pre-emulsion formation, often resulting in a broader particle size distribution compared with high-pressure homogenization. High-shear mixers provide efficient emulsification in industrial formulations [33].

##### Method Transfer for a Medium-Scale NLC Production

Upon the scale-up process, a single-factor adjustment approach was employed for the identification of impacted parameters of the NLC production, providing a rapid, cost-effective, and practical framework for industrial translation, with minimized waste [10]. This approach was employed to identify key process parameters governing successful scale-up and to support further expansion to a 5000 g batch size. The scale-up strategy focused on maintaining the particle size and uniformity of the NLCs as the production volume increased, ensuring comparability with laboratory-scale values, since particle size is a critical determinant of the performance and efficacy of absorption or permeation. The NLCs were formulated to contain 0.02% *w*/*w* curcumin (equivalent to 0.02 g per 100 g of product), representing a practically relevant concentration for topical application while minimizing the risk of irritation. In addition, curcumin-loaded nanocarrier systems have been reported to enhance skin penetration and therapeutic efficacy even at low drug loading. Therefore, the selected concentration is considered both practically relevant and pharmacologically effective for dermal delivery [3,12].

The entire process involved using HSH shear speeds of 10,000–19,000 rpm for 10–20 min and 2–4 cycles of HPH. The results from pilot-scale development (1000 g of NLCs) showed that combining HSH and HPH using a shear speed of 19,000 rpm for 20 min, followed by four cycles of industrial-scale high-pressure homogenization, resulted in particle sizes falling in the ranges of predetermined CQAs.

**Figure 8:**
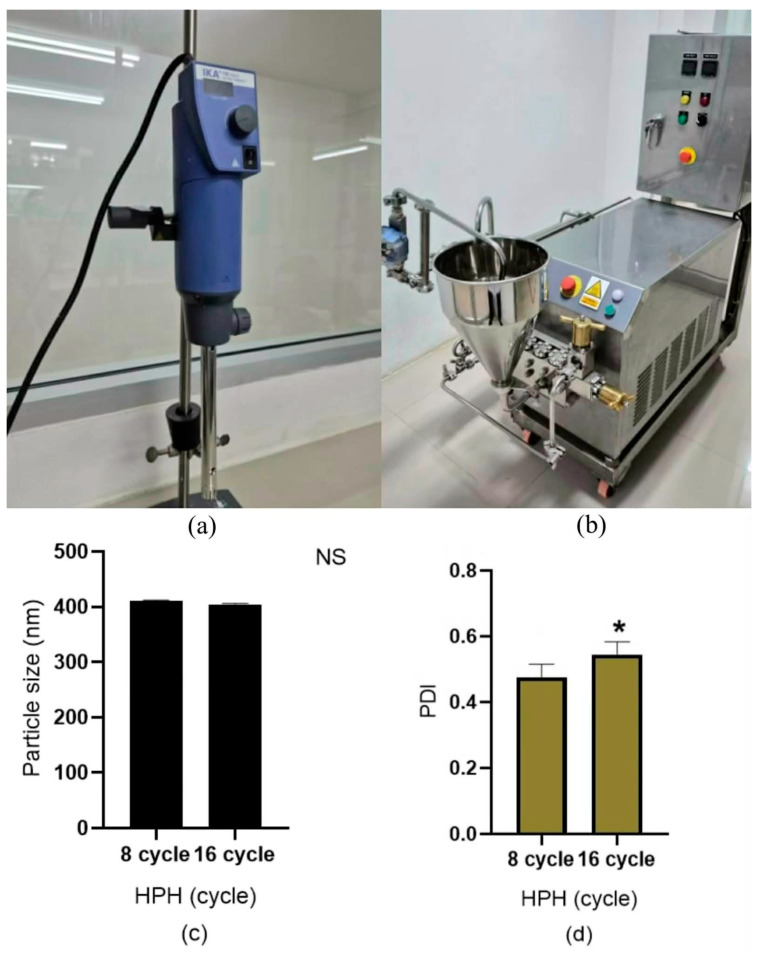
Effect of homogenization cycles on NLC properties at a production scale of 5000 g using a fixed HSH shear speed of 19,000 rpm and HSH processing time of 20 min. Sub-figure panels represent (**a**) high-shear homogenizer, (**b**) high-pressure homogenizer, (**c**) particle size, and (**d**) polydispersity index (PDI). All experiments were performed in triplicate. Asterisk (*) is significantly different at *p* < 0.05, calculated using a paired *t*-test with the SPSS program. NS indicates non-significant differences (paired *t*-test, SPSS).

Medium-scale experiments conducted under industrial conditions demonstrated that high-shear homogenization (HSH) alone was insufficient to achieve the required particle sizes, as HSH primarily contributed to pre-emulsification rather than effective nanoscale size reduction. Integration of HSH with high-pressure homogenization (HPH) (Figure 8a,b) was, therefore, required to meet the target product profile (TPP), with HPH pressure and homogenization cycle number identified as critical process parameters (CPPs). A five-fold increase in batch size (1000 to 5000 g) was evaluated while maintaining constant HSH conditions (19,000 rpm for 20 min) to isolate the effects of HPH-related CPPs. Due to the pressure limitations of industrial HPH equipment, increasing the number of cycles from the laboratory-scale condition of 4 to 8 cycles was necessary to achieve acceptable particle sizes. However, further increasing the homogenization cycles to 16 did not improve particle size reduction and, instead, negatively affected the particle size distribution, resulting in a higher polydispersity index (Figure 8c,d). These results indicate the presence of an optimal HPH operating window, beyond which excessive energy input compromises size uniformity without improving the target CQA, highlighting the importance of QbD-driven identification and control of CPPs for robust and scalable NLC production. Following scale-up production, the NLCs were incorporated into a standard cream base to yield a ready-to-use topical formulation (10 kg batch), which was subsequently subjected to clinical evaluation in human volunteers.

In addition to lipid-based nanocarriers, alternative strategies to enhance the solubility of curcumin have been reported, including micellar systems based on non-ionic surfactants such as polysorbate 80 (Tween 80). These systems can effectively solubilize curcumin within micellar structures, resulting in improved aqueous dispersibility, stability, and bioavailability. For example, polysorbate 80-based curcumin micelles have been shown to form nanosized particles (~20 nm) and significantly enhance biological performance compared with free curcumin. Such approaches are also reflected in commercially available products (e.g., micellar curcumin formulations such as Collanol^®^). Compared with these surfactant-based micellar systems, the NLC formulation developed in this study provides an alternative delivery platform with potential advantages in terms of sustained release, structural stability, and suitability for topical applications [34].

##### Optimization of CPPs of Scaled-Up Curcumin-Loaded NLCs

**Figure 9:**
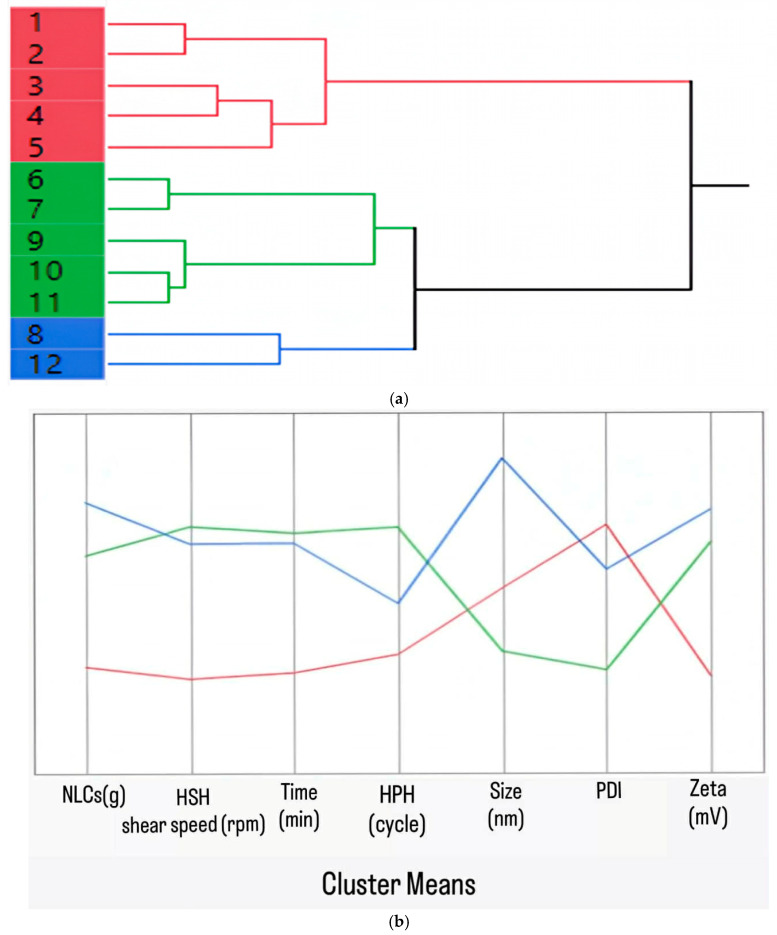
(**a**) Dendogram of lipid content and HPH conditions, considering their similarity in the CQAs’ expression, and (**b**) cluster summary. Cluster 1 (red) comprises NLCs with large particle sizes (>180 nm) and relatively high polydispersity (PDI: up to 0.73). Cluster 2 (green) exhibits the smallest particle size, approximately 150 nm, and a narrow uniform distribution (PDI: 0.2–0.3). Cluster 3 (blue) represents the largest NLCs, measuring greater than 240 nm, with a PDI of approximately 0.4.

**Figure 10:**
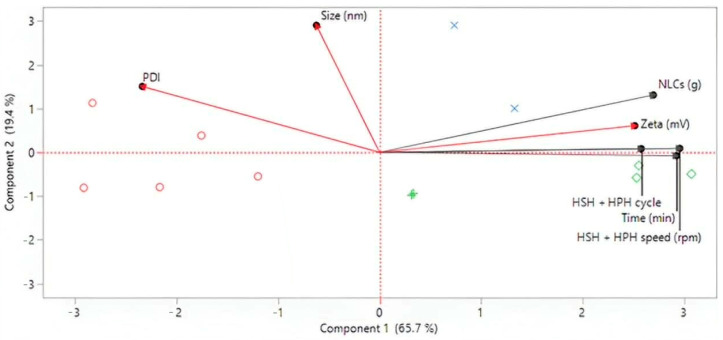
Principal component analysis (PCA) plotted along the first two axes, with the representation of the 12 formulations and the CPPs (gray lines) corresponding to physicochemical properties of curcuminoid-loaded NLCs (red lines) for the first two components.

While traditional DOE was not employed for multivariate optimization, principal component analysis (PCA) and hierarchical cluster analysis (HCA) were applied to the dataset from systematic single-factor screening (Table 1, Table 2 and Table 3; Figure 3, Figure 4, Figure 5 and Figure 6). Multivariate analysis was utilized to assess the influence of independent variables, specifically critical process parameters (CPPs), such as lipid content and HSH and HPH conditions, on dependent variables, which included critical quality attributes (CQAs) such as particle size, polydispersity index (PDI), and zeta potential. These CQAs serve as indicators of the physicochemical characteristics of curcuminoid-loaded NLCs. In particular, the CQAs are likely to affect various physicochemical properties, including loading capacity, release kinetics, stability in both aqueous and biological environments, and other in vitro and in vivo parameters. The CQAs of curcumin-loaded NLCs were defined as a minimal particle size of approximately 150 nm, PDI values in the range of 0.2–0.3, and a zeta potential of around –30 mV. The preparation trends were analyzed using hierarchical cluster analysis (HCA), which exploited the hierarchical distribution illustrated in Figure 9a,b, together with principal component analysis (PCA), represented by biplots of the first two principal components shown in Figure 10.

Hierarchical cluster analysis (HCA) was utilized to identify groups of curcuminoid-loaded NLCs with similar properties, facilitating an evaluation of the NLCs based on varying lipid content and HPH process parameters (Figure 9a). During production, evaluating the relationship between CPPs and CQAs is important. Figure 9b provides an interpretation of each cluster, confirming the similarity profile among formulations and illustrating the mean values corresponding to each variable within their respective clusters. Three clusters were identified based on the dendrogram and cluster summary. Cluster 1 (red) comprised NLCs with large particle sizes (>180 nm) and relatively high polydispersity (with PDIs of up to 0.73), which were associated with low to medium lipid concentrations and insufficient homogenization parameters, such as low speeds (10,000–12,000 rpm), short durations (10–15 min), and fewer cycles. Cluster 2 (green) exhibited the smallest particle size, approximately 150 nm, and a narrow uniform distribution (PDI: 0.2–0.3), consisting of NLCs formulated with medium to high lipid contents (500–1000 g) and a high homogenization intensity, especially high speed, i.e., 14,000–19,000 rpm, and extended durations of 15–20 min. Finally, cluster 3 (blue) represented the largest NLCs, measuring greater than 240 nm, with a PDI of approximately 0.4, which resulted from a high lipid content (1000 g) and specific HPH processing conditions (16,000 rpm for 15–20 min). The analysis indicated that increasing lipid content and optimizing HPH conditions provide curcuminoid-loaded NLCs with a small size and narrow size distribution. Furthermore, across all clusters, the zeta potential remained within a relatively stable range (approximately −26 to −36 mV), suggesting that all 12 formulations possessed good physical stability against particle aggregation.

The contributions of each CPP and CQA were analyzed across the 12 NLC formulations. The first two PCAs accounted for over 70–80% of the total variability (Figure 10), indicating variability within the data. No parameter factors were located near the center of the biplot, confirming the selected variables were significant in defining the system’s variance. The PCA demonstrated a statistical relationship between homogenization intensity and NLC particle characteristics. All CPPs and zeta potential (ZP) were associated with PC1, while particle size and PDI contributed positively to PC2. Among the CPPs, lipid content was strongly associated with the first two components (loading score: 0.43), whereas the HSH + HPH cycle (loading score: 0.49) emerged as a critical variable in explaining further variance, contributing notably to PC3. This indicated that the number of homogenization cycles exerted a distinct influence on the NLC properties that were orthogonal to the effects of speed and duration. Regarding zeta potential, HPH speed should be considered, as processing intensity may influence particle interactions during homogenization. In this study, higher HPH speed tended to reduce the absolute value of the zeta potential. Similar influences of homogenization conditions as critical process parameters in the design of ultra-small NLCs have been reported by Mendes et al. (2021) [35]. Nonetheless, it is important to note that the zeta potential values remained above −30 mV, indicating robust physical stability through electrostatic repulsion. In summary, the PCA aligns with QbD principles for data-driven design space definition, complementing the risk assessment (FMEA, Ishikawa diagram) and supporting a robust scale-up control strategy.

#### 3.3.3. Entrapment Efficiency (EE) and Loading Capacity (LC) of Curcuminoid-Loaded NLCs

**Figure 11:**
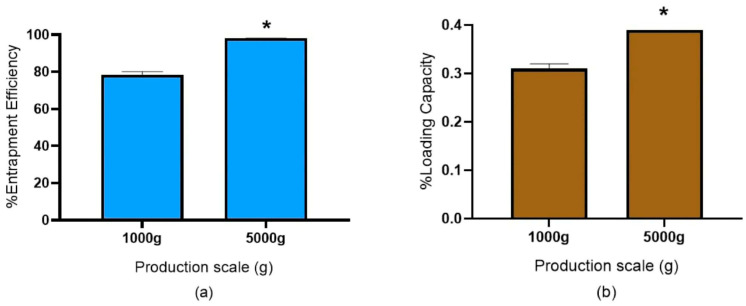
Evaluation of curcuminoid-loaded NLCs and redispersibility index. Sub-figure panels represent (**a**) entrapment efficiency and (**b**) loading capacity. All experiments were performed in triplicate. Asterisk (*) is significantly different at *p* < 0.05, calculated using a one-way paired *t*-test with the SPSS program.

The comparison of the loading capacity and retention efficiency of pilot-scale and medium-scale production is demonstrated in Figure 11. The experimental findings unequivocally demonstrate that raising the production volume using the parameters obtained from the smaller production scales studied could enhance the loading capacity and active chemical retention efficiency within NLCs. This pattern is in line with earlier research, including studies involving mRNA formulations, in which the NLC production at a larger batch size could obtain optimal nanoparticle properties, such as an increased encapsulation efficiency and loading capacity [36]. The current studies confirm that QbD process optimization can greatly increase encapsulation efficiency to as much as 90%, where the retention efficiency rises as the production scale or process concentration increases.

#### 3.3.4. QbD-Driven Scale-Up Strategy for NLC Production

**Figure 12:**
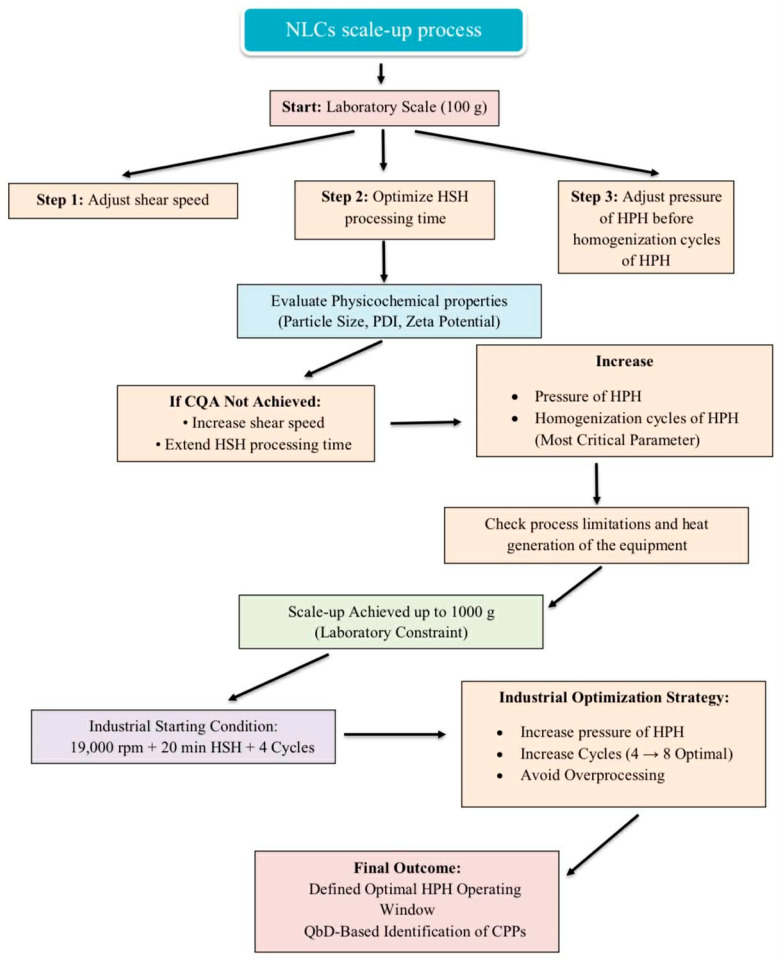
Integrated scale-up workflow for NLC production demonstrates coordinated adjustment of shear speed of HSH, HSH processing time, and homogenization cycles of HPH during batch size increase. The diagram highlights the transition from laboratory-scale optimization to industrial implementation and defines the practical HPH operating window under equipment and heat limitations.

The overall objective of this study was to develop and optimize the production process of the NLC delivery system during scale-up from laboratory scale (100 g) to a larger batch size (5000 g). The scale-up process was initiated based on the optimal formulation and processing conditions previously obtained at the 100 g laboratory scale. In accordance with Quality by Design (QbD) principles, the target product profile (TPP) was first defined, and the corresponding critical quality attributes (CQAs), including particle size, polydispersity index (PDI), and zeta potential, were identified. A risk assessment was subsequently performed to determine the potential impact of the processing variables on the defined CQAs, leading to the identification of critical process parameters (CPPs). Process optimization was conducted stepwise. Initially, the shear speed was adjusted, followed by modification of the HSH processing time. Subsequently, the number of HPH cycles was varied to determine the most suitable processing conditions. The results clearly demonstrated that both the shear speed and HSH processing time significantly influenced the physicochemical characteristics of the developed NLCs. Therefore, further optimization was performed by increasing the shear speed and extending the HSH processing time. In addition, the number of homogenization cycles was further increased to enhance system performance. However, process limitations and equipment-related heat generation restricted the maximum applicable shear speed and homogenization cycles during scale-up. Excessive thermal accumulation during processing may compromise formulation stability and limit operational feasibility. Consequently, under laboratory-scale equipment constraints, the process could only be effectively scaled up to 1000 g. For potential industrial application, the findings suggest that an appropriate starting condition would be a 19,000 rpm shear speed, a 20 min HSH processing time, and four homogenization cycles. Further optimization at an industrial scale may be achieved by gradually increasing the pressure and number of homogenization cycles, which was identified as the most critical parameter influencing system performance, followed by the HSH processing time and shear speed in Figure 12.

## 4. Conclusions

The scale-up of curcuminoid-loaded nanostructured lipid carriers (NLCs) was carried out using a two-step process comprising pre-emulsification by high-shear homogenization, followed by nanoscale size reduction via high-pressure homogenization. This combined approach is widely recognized as a robust and scalable technique for the commercial manufacture of NLCs. However, when process optimization is conducted using a design of experiments (DOE) framework, a full factorial design would require a total of 216 experimental runs per replicate, imposing an impractically high experimental burden. Such an extensive design necessitates substantial quantities of raw materials, thereby increasing both operational costs and material waste. Consequently, a systematic single-factor screening, followed by a principal component analysis (PCA) and a hierarchical cluster analysis (HCA), was adopted to enable a more efficient process optimization strategy, reducing the number of experimental runs while maintaining systematic process understanding and minimizing material consumption.

It was observed that HSH alone was insufficient to achieve effective particle size reduction, likely due to limitations in shear-induced disruption; therefore, HPH was necessary to attain the desired particle size. This experiment revealed that the development of a delivery system from laboratory to industrial settings could be compared using the experimental tables. When producing 500 g, it was possible to increase the shear speed and processing time while fixing the homogenization cycles at three cycles. Combining HSH with HPH using a shear speed of 19,000 rpm for 20 min and 4–8 cycles of industrial high-pressure homogenization resulted in particle sizes close to those obtained in the laboratory. Scale-up investigations confirmed that the homogenization speed, pressure, and number of HPH cycles significantly influenced the nanoparticle characteristics, while the integration of HSH pre-emulsification with HPH enabled reproducible semi-industrial- and industrial-scale production of uniformly distributed, nanosized NLCs, with high drug entrapment. When incorporated into a cream base, they produced a semi-solid, smooth, homogeneous yellow cream product exhibiting a herbal topical product for dermatological use. The NLC characteristics included a particle size ranging from 100 nm to 500 nm; PDI values ranging from 0.2 to 0.6; zeta potentials ranging from −25 to −40 mV; and an encapsulation efficiency ranging from 75 to 100%. Furthermore, the product exhibited physical stability during storage (6 months), with no significant phase separation or color change.

The optimized process successfully translated NLC production from the laboratory to industrial scale, yielding a physically stable topical cream with desirable physicochemical properties. Incorporation of QbD principles ensured robust process understanding, precise control within an optimized design space, and consistent product quality. Overall, a QbD-guided approach is essential for the scalable development of NLC-based drug delivery systems.

## Figures and Tables

**Table 1:** Process and formulation parameters addressed in experimental designs.

Process	Parameters	Physicochemical Properties
Pre-emulsion by HSH	Shear speed (rpm)	Particle size, PDI, and zeta potential
	Time (min)	
	Production scale	
Size reduction by HPH	Shear speed (rpm)	
	Time (min)	
	Homogenization cycles	
	Production scale	

**Table 2:** Pre-emulsion step for NLCs using high-shear homogenizer.

Run	Production Scale (g)	HSH (rpm)	HSH (min)
1	200	10,000	10
2		12,000	10
3	500	10,000	10
4		12,000	10
5		12,000	15
6		14,000	15
7		16,000	15
8	1000	16,000	15
9		16,000	20
10		16,000	20
11		18,000	20
12		19,000	20

**Table 3:** Size reduction step for NLCs using high-pressure homogenizer.

Run	Production Scale (g)	HPH (Cycle)
1	200	2
2		2
3	500	3
4		3
5		3
6		3
7		3
8	1000	3
9		3
10		4
11		4
12		4

## Data Availability

The original contributions presented in this study are included in the article. Further inquiries can be directed to the corresponding author.
